# Maternal Protein Malnutrition Does Not Impair Insulin Secretion from Pancreatic Islets of Offspring after Transplantation into Diabetic Rats

**DOI:** 10.1371/journal.pone.0030685

**Published:** 2012-02-27

**Authors:** Renato Chaves Souto Branco, Júlio Cezar de Oliveira, Sabrina Grassiolli, Rosiane Aparecida Miranda, Luiz Felipe Barella, Rodrigo Mello Gomes, Luiz Augusto Bataglini, Rosana Torrezan, Clarice Gravena, Paulo Cezar de Freitas Mathias

**Affiliations:** 1 Laboratory of Secretion Cell Biology, Department of Cell Biology and Genetics, State University of Maringá, Maringá, Brazil; 2 Laboratory of Biology Development, Department of Biology, State University of Ponta Grossa, Ponta Grossa, Brazil; University of Chicago, United States of America

## Abstract

Pancreatic islets from adult rats whose mothers were protein restricted during lactation undersecrete insulin. The current work analyzes whether this secretory dysfunction can be improved when the pancreatic islets are grafted into hyperglycemic diabetic rats. Two groups of rats were used: the adult offspring from dams that received a low protein diet (4%) during the initial 2/3 of lactation (LP) and, as a control, the adult offspring from dams that consumed a normal protein diet (23%) during the entire period of lactation (NP). Islets from NP- and LP-rats were transplanted into diabetic recipient rats, which were generated by streptozotocin treatment. The islets were transplanted via the portal vein under anesthesia. The fed blood glucose levels were monitored during the 4 days post-transplantation. Transplanted islets from LP-rats (T LP) decreased the fed glucose levels of diabetic rats 34% (21.37±0.24 mM, p<0.05); however, the levels still remained 2-fold higher than those of the sham-operated controls (6.88±0.39 mM, p<0.05). Grafts with NP-islets (T NP) produced the same effect as the LP-islets in diabetic rats. The high fasting blood glucose levels of diabetic rats were improved by the transplantations. Islet grafts from both rat groups recovered 50% of the retroperitoneal fat mass of the diabetic rats (0.55±0.08 g/100 g of body weight for T NP and 0.56±0.07 g/100 g of body weight for T LP, p<0.05). Because pancreatic islets from both the NP- and LP-rats were able to regulate fasting blood glucose concentrations in hyperglycemic rats, we propose that the altered function of pancreatic islets from LP-rats is not permanent.

## Introduction

Some evidence suggests that the etiology of obesity is not only related to overnutrition but also to food restriction during early life [1]–[4]. There are numerous studies showing that nutrient deprivation of the fetus increases the risk of developing metabolic diseases in adult life [5], [6]. The mother's condition during pregnancy and lactation is very important and affects the offspring's central nervous system development, particularly that of rodents, and constitutes a sensitive window during which nutritional insults may cause metabolic disturbances in the offspring's adult life [7], [8]. Overnutrition during lactation provokes obesity and hyperinsulinemia, among other hallmarks of the metabolic syndrome, whereas undernutrition permanently decreases body weight and insulin levels. Moreover, it has been shown that pancreatic islets from adult rats that were fed during lactation by protein-restricted dams undersecrete insulin [8]–[13]. This metabolic impairment is a long-term effect, as it remains in adult life even when dietetic recovery is allowed [13]. It has been suggested that early nutritional injuries, which cause metabolic programming, change gene expression; however, it is not a mutation-dependent phenomenon [14]. The beta cell size, proliferation and vascularization are reduced in rats that were protein malnourished neonatally [15], [16]. Injuries occurring in critical periods of development are associated with permanent and progressive changes in gene expression. By analyzing islets isolated from male rats at 7 weeks old that were growth restricted *in utero*, researchers found dysregulated cytosine methylation at 1400 loci, which were preferentially at conserved intergenic sequences and near genes regulating islet beta cell proliferation, vascularization, insulin secretion and cell death [17]. Recently, it was shown that pancreatic islet malfunction in rats with high-fat diet-induced obesity is transmitted by the father to female offspring [18]. Considering that malfunctions of beta cells in rats that were maternally protein restricted occurred during a metabolism-programming milieu, the conditions of the beta cell could improve for insulin release once they are in a different metabolic environment. The purpose of the current work was to analyze whether the secretory dysfunction of pancreatic islets from adult rats that were submitted to a maternal protein deficit during lactation can be improved when grafted into hyperglycemic diabetic rats.

## Results

### The effect of maternal protein restriction on adult offspring

LP-rats were 14.6% lighter and 8% shorter than NP rats, (p<0.05). An early maternal low-protein diet treatment led to a 23.5% reduction in fat tissue accretion in LP-rats compared to NP-rats, (p<0.05).

The LP-rats presented 39% lower plasma insulin levels when compared with NP animals, (p<0.05), while glycemia was unchanged between the groups, as shown in table 1.

**Table 1:** The effect of maternal protein restriction on adult rats.

	NP	LP
Body weight (g)	376.32±6.43	321.15±4.78*
Body length (cm)	22.63±0.35	20.82±0.45*
Retroperitoneal fat (g/100 g BW)	1.06±0.05	0.81±0.07*
Fasting glycemia (mmol/L)	4.97±0.03	5.10±0.11^NS^
Fasting insulinemia (ng/mL)	0.18±0.02	0.11±0.02*

Data represent the mean ± SEM obtained from 30–45 animals per experimental group. Significant differences were determined using Student's t-test with *p<0.001.

### The effect of maternal protein restriction on glucose homeostasis of adult offspring

**Figure 1:**
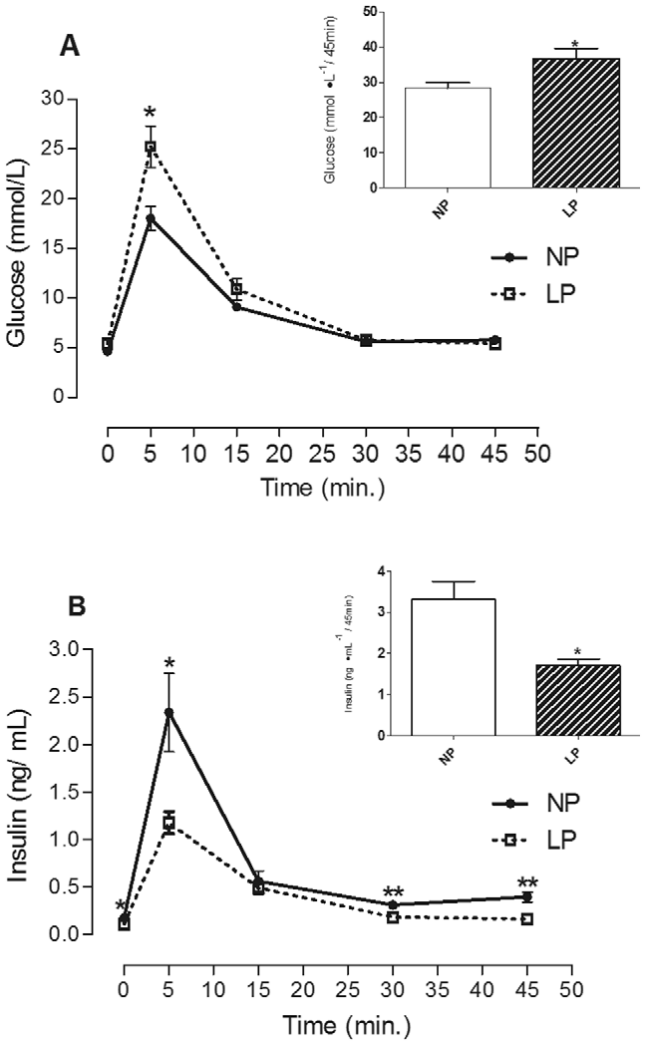
The effects of maternal protein restriction on the glucose and insulin homeostasis of adult rats. Data represent the mean ± SEM of a minimum of 7 rats per experimental group. **A**) glucose concentrations for the entire ivGTT timecourse. Inset shows the glycemia AUC for the ivGTT. **B**) insulin concentrations for the entire ivGTT timecourse. Inset shows the insulinemia AUC for the ivGTT timecourse (*p<0.05, **p<0.01 by Student's t-test).

Although LP-rats were normoglycemic after 45 minutes of an ivGTT, the plasma glucose concentrations were higher than the NP-rats 5 minutes after the glucose load, as presented in figure 1A. Calculations of the area under the curve (inset of figure 1A) show that total glycemia was 28.7% higher in LP-rats than in NP ones, (p<0.05). Figure 1B shows that the low insulin concentrations of fasting LP-rats remained lower that the NP-rats during the entire ivGTT timecourse (except at 15 minutes). As represented by the area under the insulinemic curve (inset of figure 1B), the LP-rats showed reduced insulinemia compared to the NP-rats, (p<0.05).

### Glucose-induced insulin secretion in isolated pancreatic islets

**Figure 2:**
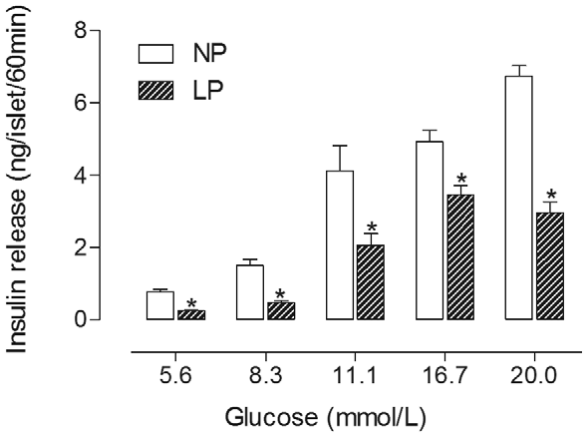
Glucose-induced insulin secretion from isolated pancreatic islets. The graph shows the effect of maternal protein restriction on insulin secretion from islets that were stimulated by glucose. The bars represent the mean ± SEM of 6 NP and 7 LP rats (*p<0.05 by Student's t-test).

Figure 2 shows that islets from the LP-rats release less insulin when stimulated by different glucose concentrations compared with islets from the NP-rats. For instance, the release of insulin from LP-islets compared to NP-islets was 3-fold less in response to 5.6 mmol/L glucose and to 8.3 mmol/L glucose, 2-fold less in response to 11.1 mmol/L glucose, 1.5-fold less in response to 16.7 mmol/L glucose and 2.25-fold less in response to 20.0 mmol/L glucose, (p<0.05).

### The effect of islet grafts on the fasting and non-fasting glycemia of diabetic rats

**Figure 3:**
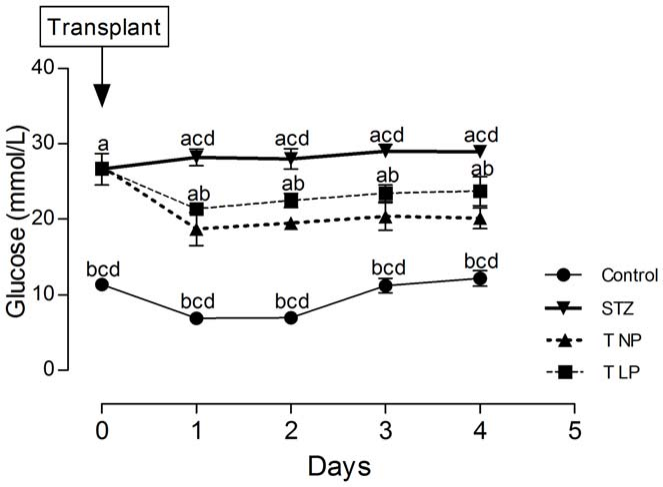
The effect of the islet grafts on the non-fasting glycemia of diabetic rats. The graph shows the islet grafts' effects on the fed blood glucose concentrations during the 3 days after the islets' transplantation. Data represent the mean ± SEM of 7 to 8 rats per experimental group. The following letters represent significant differences between the groups on the indicated days: (a) control, (b) diabetic (STZ), (c) T NP and (d) T LP (p<0.05 by one-way ANOVA).

**Figure 4:**
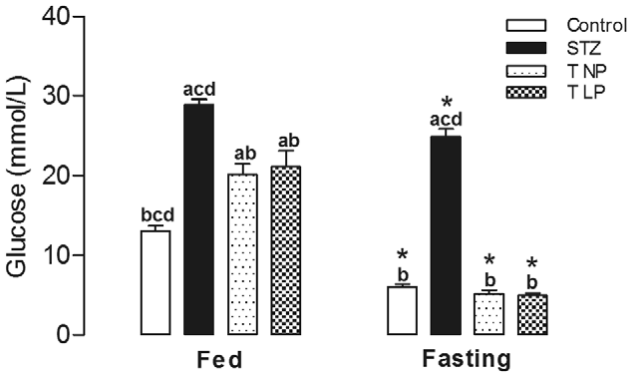
The effect of the islet grafts on the fasting and non-fasting glycemia of diabetic rats. The graph shows the effect of grafted islets on fed and fasting plasma glucose levels. Data represent the mean ± SEM of 7 to 8 rats per group. The following letters represent significant differences between all fed groups and all fasting groups by one-way ANOVA where p<0.05 is indicated by (a) control, (b) diabetic (STZ), (c) T NP and (d) T LP.

Before the islet graft, fed diabetic rats presented 2.2-fold higher glucose concentrations that the non-diabetic control animals, (p<0.05), as shown in figure 3. This severe hyperglycemia did not change over a period of 4 days. One day after the grafts of islets from NP- or LP-rats, the diabetic animals with transplanted islets presented a 30% decrease in their fed plasma glucose levels, (p<0.05); however, their glycemia was 2.3-fold higher than the non-diabetic control rats, as shown in figure 3. In the subsequent 3 days, the glycemia of both groups of diabetic rats with islet grafts were unchanged. On the fifth day after transplantation, the fasting glycemia of the diabetic rats was 5-fold higher compared to non-diabetic control rats, (p<0.05), as shown in figure 4. In contrast, both groups of transplanted animals presented fasting plasma glucose levels 79% lower than the diabetic animals, (p<0.05). Figure 4 also shows that the hyperglycemia present in fed animals decreased in the fasting condition by 14% for diabetic rats, 54% for non-diabetic controls and 75% for both of the grafted diabetic rat groups, (p<0.05).

### Insulin secretion of grafted diabetic rats

**Figure 5:**
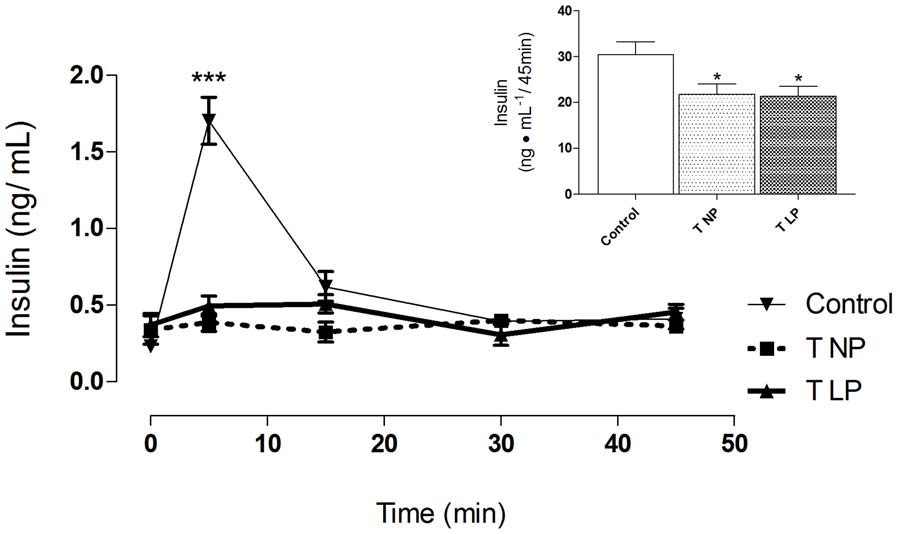
Insulin secretion in the grafted diabetic rats. Data represent the mean ± SEM of 7 to 8 rats per experimental group. The inset represents the area under the insulinemic curve during the entire ivGTT timecourse (*p<0.05 by Student's t-test). The insulin levels in the STZ rats were not detectable by this method.

Grafted rats with islets from either the NP- or the LP-rats did not show the transient increase in plasma insulin concentrations that was observed in the non-diabetic control rats, as shown in figure 5; however, by calculating the area under the insulinemic curve (inset), grafted animals secreted up to 70% of the amount of insulin released by the non-diabetic rats, (p<0.05). Blood insulin levels of the diabetic control rats were not detectable using the method described in the current work.

### The effect of islet grafts on retroperitoneal fat accumulation in diabetic rats

**Figure 6:**
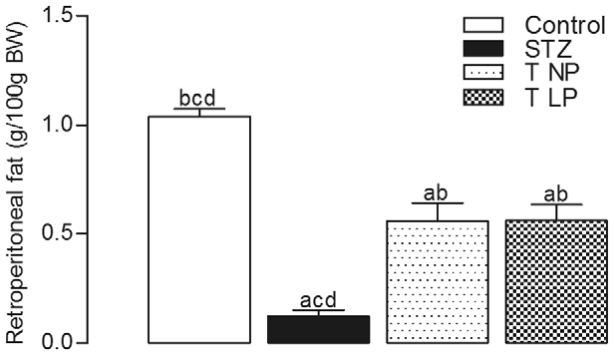
The effect of the islet grafts on the retroperitoneal fat accumulation of diabetic rats. The graph shows the effect of grafted islets on the retroperitoneal fat pad. Data represent the mean ± SEM of 7 to 8 rats per group. The following letters represent significant differences between all experimental groups by a one-way ANOVA with p<0.05 indicated by (a) control, (b) diabetic (STZ), (c) T NP and (d) T LP.

Although the treatment with streptozotocin (STZ) causes hyperglycemia, fat deposition decreased 10-fold in the retroperitoneal fat pads of diabetic rats compared with those of the non-diabetic rats, (p<0.05). Islets grafts caused a 6-fold increase in retroperitoneal fat accumulation when compared to non-grafted diabetic rats, (p<0.05); however, when compared to non-diabetic rats, the fat tissue accumulation was still 50% lower in the grafted animals with islets from either group, (p<0.05), as shown in figure 6.

## Discussion

In the current work, maternal protein restriction during lactation provoked a metabolic impairment in adult rats, including weak glucose-induced insulin release, as was previously described in the literature [8], [10], [12], [13], [19]–[25]. To release insulin, the beta cell acts as a metabolic sensor with the glucose degradation rate as the main intracellular signal to trigger exocytosis of insulin granules [26], [27]. There was no difference in the insulin content of islets from the offspring of protein-malnourished rats [12]; moreover, no alterations in the transduction signals involved in coupling insulin-stimulated secretion to metabolism were found in beta cells from the offspring of protein-malnourished rats [12]. However, alternative mechanisms involved in the glucose-induced insulin release process and beta cell malfunctions, such as the K^+^ATP-independent pathway [28] or, as recently suggested, the AMPK pathway [29], should be examined. It has been demonstrated that protein restriction in maternal nutrition causes irreversible metabolic changes, which includes beta cell dysfunction [13]; in addition, these changes are not related to gene alterations but possibly to changes in gene expression and/or epigenetic control [30]. Metabolic imprinting by early protein-depleted maternal feeding leads to beta cell malfunction that programs the entire organism towards a distinct metabolic regulation. In this metabolic environment, where the activity of the autonomic nervous system is altered [10], [13], insulin secretion is affected. Furthermore, in another metabolic condition, the secretory response of beta cells from the offspring of protein malnourished rats could be improved. The current work shows that grafts of LP-islets reduced the fed-state hyperglycemia of diabetic rats but did not fully return it to the level of the control rats. Although the grafts of NP-islets did not completely correct the hyperglycemia of diabetic rats [31], it could be argued that the grafts were recent, and differences in the transplanted rats might be observed over time. It could be hypothesized that grafted LP-islets would not decrease the high glycemia of diabetic recipient rats while NP ones would be more effective; however, this was not observed. It has been shown that a simple islet transplantation of a short duration, up to 5 days, and independent of islet number did not correct the hyperglycemia of STZ diabetic rats; however, long-term islet grafts promote fed-state normoglycemia depending on the site of the transplantations [31]–[33].

While glucose is the major physiological signal to induce insulin secretion, other fuel compounds, such as amino acids, fatty acids and many others metabolites, that stimulate beta cell metabolism also induce insulin secretion [34]. Beta cells receive many neural terminals, which release neuropeptides and neurotransmitters that also participate in the regulation of insulin secretion. For instance, neurotransmitters, such as acetylcholine and noradrenaline, are able to potentiate or inhibit glucose- and nutrient-induced insulin secretion, respectively [35]. It has been demonstrated that after 5 days, grafted islets show innervations, which allow for the amelioration of glycemic regulation [36]. The lack of a neuronal influence through a short-term (4 day) islet graft, as was studied in the current work, might have contributed to the weak glucose-induced insulinotropic effect observed for the grafted islets; alternatively, it has been shown that the islets' number and mass decrease on subsequent days after transplantation [37]–[39]. Whereas islet grafts did not restore the fed hyperglycemia of diabetic rats to the level of control rats, transplantations with islets from either group were able to completely normalize fasting hyperglycemia. An improved fed glycemia in diabetic rats that were grafted was observed during the first days after an islet transplantation into the capsule of the right kidney or liver [31], [32]. Although even freshly isolated LP-islets undersecrete insulin upon stimulation by low glucose concentrations, which are similar to the plasma glucose concentrations found during overnight fasting, the grafted LP-islets were able to secrete insulin in quantities sufficient to control glycemia. It must be emphasized that LP-islets also released very low insulin levels compared with the secretion from NP ones when stimulated by high glucose concentrations, as was observed in the diabetic rats. Although LP-islets undersecrete insulin, they were able to reduce glycemia by the same magnitude as NP-islets when grafted into hyperglycemic diabetic rats. LP-islets that were transplanted into diabetic rats were able to maintain blood insulin levels and to stimulate fat tissue accumulation to partially recover the fat tissue stores, which are depleted when diabetes is established [40]. Insulin has a lipogenic effect in some tissues, including adipose tissue, through the activation of genes involved in lipid metabolism and glucose homeostasis [41]. The results from the current work suggest that metabolic programming due to perinatal protein restriction provokes long-term changes in pancreatic beta cells; however, it might be possible for the beta cells to recover their function. Understanding the precise mechanism that programs and deprograms metabolism, which results in malfunctions of the beta cells in early metabolically imprinted organisms, may be crucial in reducing the world-wide epidemic of metabolic diseases, such as obesity and diabetes.

## Materials and Methods

### Animal and dietary treatments

Female Wistar rats were fed a normal laboratory diet with 23% protein content (Nuvital®, Curitiba, Brazil) throughout pregnancy. After giving birth, the rats were distributed into two groups, with each lactating dam housed with six pups. Because gender differences in insulin levels and glucose tolerance have been observed in some early poor-protein feeding studies [24], only male offspring were used in these experiments. In the normal protein (NP) group, all dams received a normal protein (23%) diet *ad libitum* during lactation, whereas the low protein (LP) group received a 4% protein diet containing the same number of calories as the normal protein diet (table 2), as described previously [20]. In the LP group, all dams received a low protein diet *ad libitum* during the first 14 days of lactation before being returned to the normal diet for the remainder of the lactation period. At 21 days of age, the pups were weaned and then fed a normal diet for 60 days, which was when the analyses were performed. Throughout the experimental period, the rats were kept under controlled temperature (22±2°C) and light (07:00 to 19:00 h) conditions with water and food provided *ad libitum*.

**Table 2:** The composition of the normal- and low-protein diets.

Components (g)	Control diet (23%)	Low-protein diet (4%)
Sucrose	12.72	20.00
Cornstarch	52.75	64.25
Casein (88% protein)	23.33	4.55
Mixture of mineral salts *	3.20	3.20
Vitamin mixture *	1.60	1.60
Soybean oil	4.80	4.80
Fish oil	1.60	1.60
**Total**	**100.00**	**100.00**

*The mixtures of salts and vitamins that were used in the manufacturing of the diet followed the recommendations of the AIN 93 [13]. Values are presented as g/100 g of diet.

The Ethical Committee for Animal Experiments for the State University of Maringá, which adheres to Brazilian Federal Law, approved the protocols (059/2005-CEA).

### The effects of maternal protein restriction on biometric parameters of adult rats

After an overnight fast, 81-day-old adult rats from both the NP and LP groups were weighed, and their nose-anal lengths were taken. Subsequently, the rats were anesthetized by an intraperitoneal injection of sodium pentobarbital (45 mg/kg of body weight (BW)) and killed by cervical dislocation. The retroperitoneal fat pads were removed and weighed. The mass of this tissue was used as a simple, reliable way to estimate the body fat in the rodents [42].

### Intravenous glucose tolerance test (ivGTT)

Under ketamine and xylazine anesthesia (5.5 and 0.8 mg/100 g BW, respectively), a silicone cannula was implanted into the right jugular veins and stabilized on the dorsal regions of the necks of rats from both groups. The cannula was previously treated with a heparin/saline solution (50 IU heparin/mL of 0.9% saline solution) to prevent blood clots. After a 12-h fast and without anesthesia, a glucose load (1 g/kg BW) was infused in the animals through the cannula. Blood samples were collected immediately before (time 0) and at 5, 15, 30 and 45 min after the glucose load. Plasma obtained from the blood samples was stored at −20°C for further analysis of the glucose dosages and insulin concentrations. The areas under the glycemic and insulinemic curves (AUCs) from the ivGTT were calculated.

### Induction of diabetes in the rats

Diabetes was induced by STZ (50 mg/kg BW i.v.) in NP 81-day-old rats. During the 4 days following the STZ injection, blood samples were collected once every morning from the rats' tails to measure glucose levels using a commercial kit (*Gold* Analisa®). Plasma glucose concentrations from 22 to 34 mM were used to define rats as diabetic. These animals were used as islet graft recipients 5 days after the STZ injections.

### Isolation of pancreatic islets

Isolation of pancreatic islets from the rats was performed as previously described [9] with adaptations. Briefly, rats from the NP and LP groups were anesthetized (sodium pentobarbital, 45 mg/kg BW), killed by cervical dislocation and then had their abdominal walls cut open. Subsequently, 8 mL of Hank's buffered saline solution (HBSS) containing collagenase type XI (1 mg/mL, Sigma Chemical Co., St. Louis, MO) was injected into the common bile duct of each rat. The pancreas, swollen with the collagenase solution, was quickly excised and incubated at 37°C in a plastic culture bottle for 11–12 min for an LP pancreas and 18–19 min for an NP pancreas. The suspension was then filtered through a 0.5 mm metal mesh and subjected to 5 continuous washes with HBSS, which contained 0.12% bovine serum albumin fraction V (BSA) and 5.6 mM glucose. Islets were collected with the aid of a microscope and washed 3 times with HBSS. One batch of islets was used to test the insulin secretory response to glucose, and another batch was used for transplantation. For each graft, 1000–1200 islets were resuspended in 0.5 mL of cold HBSS solution and stored until just before transplantation.

### Glucose insulinotropic effect

Batches of 4 islets were pre-incubated for 60 min in 1 mL of normal Krebs solution containing 20 mM Hepes (pH 7.4) and 5.6 mM glucose. After this period, the islets were submitted to different concentrations of glucose: 5.6, 8.3, 11.1, 16.7 and 20 mM for another 60 min of incubation. During the pre-incubation and incubation periods, the batches of islets were maintained in a gassed atmosphere with O_2_/CO_2_ (95/5%). Samples during the incubation were taken, frozen and stored for further measurements of secreted insulin.

### Islet transplantations

Grafts were performed as described previously [31], [43]. Diabetic rats were anesthetized with ketamine and xylazine, and the upper 2/3 of the abdomen was opened by a midline incision. The liver was held in place with cotton buds and gauze swabs. Using a microscope, the left branch of the portal vein was identified and exposed by dissection. Islet suspensions from NP-rats (T NP) or LP-rats (T LP) were gently injected into the portal vein. Immediately after the injection of the islets, coagulant sponges were applied to the site of injection for 1 to 2 min to avoid hemorrhaging. The abdomen was closed, and the rats were given postoperative analgesia. Of the 10 grafted rats in each group, three animals died from excessive bleeding during the postoperative period. The same surgery was also performed on a batch of diabetic (STZ) and NP (Control) rats.

### Graft monitoring

During the 4 mornings after the transplantations, blood samples were collected from the tips of the tails of fed animals to measure plasma glucose levels. After a 12 h fast on the fifth day after the islet transplantation, a batch of grafted rats were submitted to anesthesia (ketamine and xylazine) and killed by cervical dislocation. The retroperitoneal fat pads were isolated and weighed to estimate body fat mass. Blood samples were used to measure fasting plasma glucose concentrations.

### Intravenous glucose tolerance test (ivGTT) after transplantation

Five days after the islet transplantation, a separate batch of rats was fasted for 12 hours prior to an ivGTT being performed, as previously described. The plasma obtained from blood samples was stored at −20°C for a later analysis of the glucose dosages and insulin concentrations.

### Measurement of glucose and insulin concentrations

The glucose concentrations were measured by the glucose oxidase method [44] using a commercial kit (*Gold* Analisa®), and the concentrations of insulin were determined by an iodine-125-labeled insulin radioimmunoassay with human insulin as the standard and an antibody against rat insulin [45].

### Statistical analysis

Results are given as the mean ± SEM and were analyzed by Student's t-test or a variance analysis (ANOVA) followed by Bonferroni t-test. P values less than 0.05 were considered statistically significant. Tests were performed using GraphPad Prism version 5.0 for Windows (GraphPad Software Inc, San Diego, CA, USA).
